# The Oncotype DX® 21-gene recurrence score and clinical outcomes in hormone receptor-positive, HER2-negative male breast cancer: a scoping review

**DOI:** 10.1007/s12094-025-04110-w

**Published:** 2025-11-23

**Authors:** Silvia Mezi, Giulia Pomati, Antonella Chiavassa, Valentina Silvestri, Laura Ottini

**Affiliations:** 1 Department of Radiological, Oncological and Pathological Sciences, Sapienza University of Rome, Rome, Italy; 2 Clinical Oncology Unit, Istituto Neurotraumatologico Italiano (I.N.I), Grottaferrata, Italy; 3 Department of Molecular Medicine, Sapienza University of Rome, Rome, Italy

**Keywords:** Oncotype DX 21-gene RT-PCR, Recurrence score, Molecular characterization, Adjuvant chemotherapy, Male breast cancer

## Abstract

**Purpose:**

Male breast cancer (MBC) is a rare malignancy that remains significantly underrepresented in clinical trials. As a result, the clinical utility of Oncotype DX (ODx) 21-gene recurrence score (RS) in men has not been well established. This scoping review aims to systematically examine the current literature regarding the prognostic and predictive role of the ODx in MBC, identify relevant factors that may influence its interpretation, and highlight key research gaps that may hinder its appropriate clinical application in male patients.

**Methods:**

A comprehensive literature search was conducted in PubMed and Scopus databases for studies published from 2010 onward. Studies reporting RS results, based on the ODx 21-gene RT-PCR, along with associated clinical outcome, were included.

**Results:**

Three studies evaluating RS in relation to clinical outcomes in MBC met the inclusion criteria. A higher proportion of MBC patients exhibited RS ≥ 31 compared to female breast cancer cohorts. RS showed significant associations with clinical outcomes, including higher mortality rates in high-risk MBC patients. However, in node-negative cases, RS did not retain independent prognostic value. Although RS was prognostically significant in both sexes, outcomes tended to be worse in MBC, particularly in the intermediate- and high-risk groups, where hazard ratios associated with chemotherapy benefit changed minimally after treatment adjustment.

**Conclusion:**

The prognostic value of RS in MBC appears evident; however, sex-specific thresholds may be necessary to optimize treatment stratification. Given current data limitations, male-inclusive clinical trials are needed to elucidate the predictive value of RS in men and improve personalized treatment strategies.

## Introduction

Male breast cancer (MBC) is a rare disease, accounting for approximately 1% of all new breast cancer (BC) diagnoses [1, 2]. Compared to female breast cancer (FBC), MBC is typically diagnosed at an older age, with a peak incidence around 70 years, and at more advanced clinical stages, frequently involving lymph node metastasis, which may contribute to poorer clinical outcomes [3–5]. Histologically and molecularly, MBC is predominantly characterized by hormone receptor-positive (HR +), HER2−negative (HER2−) tumors, with consistently high estrogen receptor (ER) expression and low HER2 levels.

Environmental factors, such as obesity, and an elevated estrogen-to-androgen ratio, along with genetic predispositions, such as *BRCA1/2* pathogenic variants (PVs) and family history, contribute significantly to MBC etiology [1, 2, 5–7]. Approximately 10% of MBC cases carry germline PVs in *BRCA2*, and up to 20% have a positive family history of BC [7, 8].

Molecular profiling studies have further highlighted the distinct biology of MBC. Retrospective analyses using PAM50 have demonstrated a predominance of luminal B subtypes [9]. In addition, genomic profiling by *Johansson *et al*.* identified two clusters, termed ‘Male Complex’ and ‘Male Simple’ highlighting both shared and unique features relative to FBC [10]. These findings support the view of MBC as a biologically distinct entity, with important implications for clinical management [11–13].

Despite these differences, current treatment strategies for MBC are largely extrapolated from clinical trials conducted in women. The standard approach of early-stage MBC includes surgery followed by endocrine therapy, with adjuvant chemotherapy considered based on recurrent risk [14–16].

In recent years, genomic assays have emerged as valuable tools for refining prognosis and guiding adjuvant chemotherapy decisions. The 2016 American Society of Clinical Oncology (ASCO) guidelines endorsed the use of several genomic tests, including Oncotype DX (ODx), MammaPrint, Prosigna, EndoPredict, and Breast Cancer Index [BCI], for risk stratification in early stage HR +/HER2− BC [17].

Among these, ODx is the most widely adopted assay. It is a 21-gene RT-PCR-based test that provides a recurrence score (RS), which integrates expression data from 16 cancer-related genes involved in hormone response, proliferation, and invasion, along with 5 reference genes. Of these, the proliferation module contributes most significantly to the final score [18–23].

The ODx-RS estimates the 10-year risk of distant recurrence and the potential benefit from adjuvant chemotherapy in patients with early stage HR +/HER2−, node-negative (N0) or limited node-positive (N1, 1–3 nodes) disease. The RS has been validated in large clinical trials, including the National Surgical Adjuvant Breast and Bowel Project’s B-20 (NSABP-B-20) study and the TAILORx (Trial Assigning Individualized Options for Treatment) study, which confirmed its predictive and prognostic relevance in women with early-stage BC [18–23]. The Rxponder trial later demonstrated that postmenopausal women with 1–3 positive nodes and low RS might safely avoid adjuvant chemotherapy, while premenopausal women with similar RS benefit significantly from it [24]. However, male patients have been consistently underrepresented in these clinical trials, and men are largely excluded from ODx validation studies. Consequently, the clinical utility of ODx specifically in male patients remain unclear. To date, only a few studies have evaluated ODx specifically in men, and the use of genomic testing in MBC remains limited, thereby restricting access to personalized treatment in this population [25–27].

Preliminary data comparing RS distribution between MBC and FBC suggest broadly similar patterns. However, only a few studies have correlated RS categories with clinical outcomes in men, such as recurrence-free survival (RFS) and overall survival (OS) [28–30]. Importantly, there is no consensus regarding the predictive value of RS for chemotherapy benefit in MBC, nor on whether standard RS cutoffs, validated in women, are applicable to male patients.

This scoping review aims to consolidate the available evidence on the 21-gene ODx RS in HR +/HER2− MBC, focusing on its prognostic and predictive roles. The objective is to support evidence-based clinical decision-making and promote more personalized therapeutic strategies for men with luminal-like breast tumors.

Specifically, this review addresses the following open questions: a) are RS thresholds validated in FBC equally prognostic in MBC, or are sex-specific cutoffs needed? b) Do age and lymph node status influence RS interpretation in MBC as they do in FBC? c) What is the potential benefit of adjuvant chemotherapy across RS risk groups in male patients?

## Material and methods

### Search terms

The review protocol reported here was defined by all authors in accordance to the Preferred Reporting Items for Systematic reviews and Meta-Analyses extension for Scoping Reviews (PRISMA-ScR guidelines), to ensure the highest quality of their work [31]. The search was conducted using the PubMed and Scopus databases. It was limited to studies published from 2010 onward, as the use of the RS was endorsed by the ASCO and the National Comprehensive Cancer Network (NCCN) guidelines in 2007 and 2008, respectively [32, 33]. The search strategy was based on the following terms: ‘Oncotype DX,’ ‘adjuvant chemotherapy,’ ‘21-gene RT-PCR,’ ‘molecular characterization,’ ‘recurrence score,’ and ‘male breast cancer.’ All of the aforementioned terms were combined using operators such as ‘AND’ and ‘OR.’ All identified abstracts were screened for inclusion by two members of the review team (GP and AC) under the supervision of a senior author (SM). The selected abstracts were thoroughly assessed and included in the analysis based on the predefined inclusion criteria.

### Eligibility assessment

The authors conducted an eligibility assessment of all full-text articles that were considered for the final analysis. In the absence of randomized clinical trials including MBC, retrospective studies, including cohort studies and case–control studies that reported the RS—defined by a 21-gene RT-PCR performed in male patients—were included in the review if they associated RS with oncological outcomes. Only full-text articles written in English were included. The authors excluded all meta-analyses and systematic or narrative reviews, as well as studies employing genomic analysis methods other than RT-PCR. Articles were also excluded from the scoping review if they were editorials, commentaries, letters, news articles, case reports, or otherwise irrelevant studies.

### Data extraction and presentation

Two reviewers (GP and AC) recorded relevant data from the selected papers using a standardized data extraction form [34]. In the event of conflicting data, one of the senior authors reviewed the study for further discussion and provided assistance in determining the inclusion of data.

The following data were extracted from the included studies: publication year, first author, title, study type, country, sample size (defined as the analyzed population), patients’ inclusion criteria, mean or median age of patients at the time of cancer diagnosis, tumor stage at diagnosis, type of adjuvant treatment administered, ODx-RS values and cutoffs, outcome measures, follow-up period, univariate and multivariate results.

Data were charted using a tabulated format and pie charts, and summarized as a narrative review.

### Risk of bias assessment

Two independent reviewers (G.P. and V.S.) performed quality assessments of the studies included in the analysis using the Risk of Bias in Non-randomized Studies of Exposure (ROBINS-E), which provides a structured approach to evaluate the risk of bias in observational epidemiological studies [35]. The Robvis visualization tool web app was used to create traffic light plots reflecting the domain-level and overall assessments for each individual study [36].

## Results

### Study selection

**Fig. 1:**
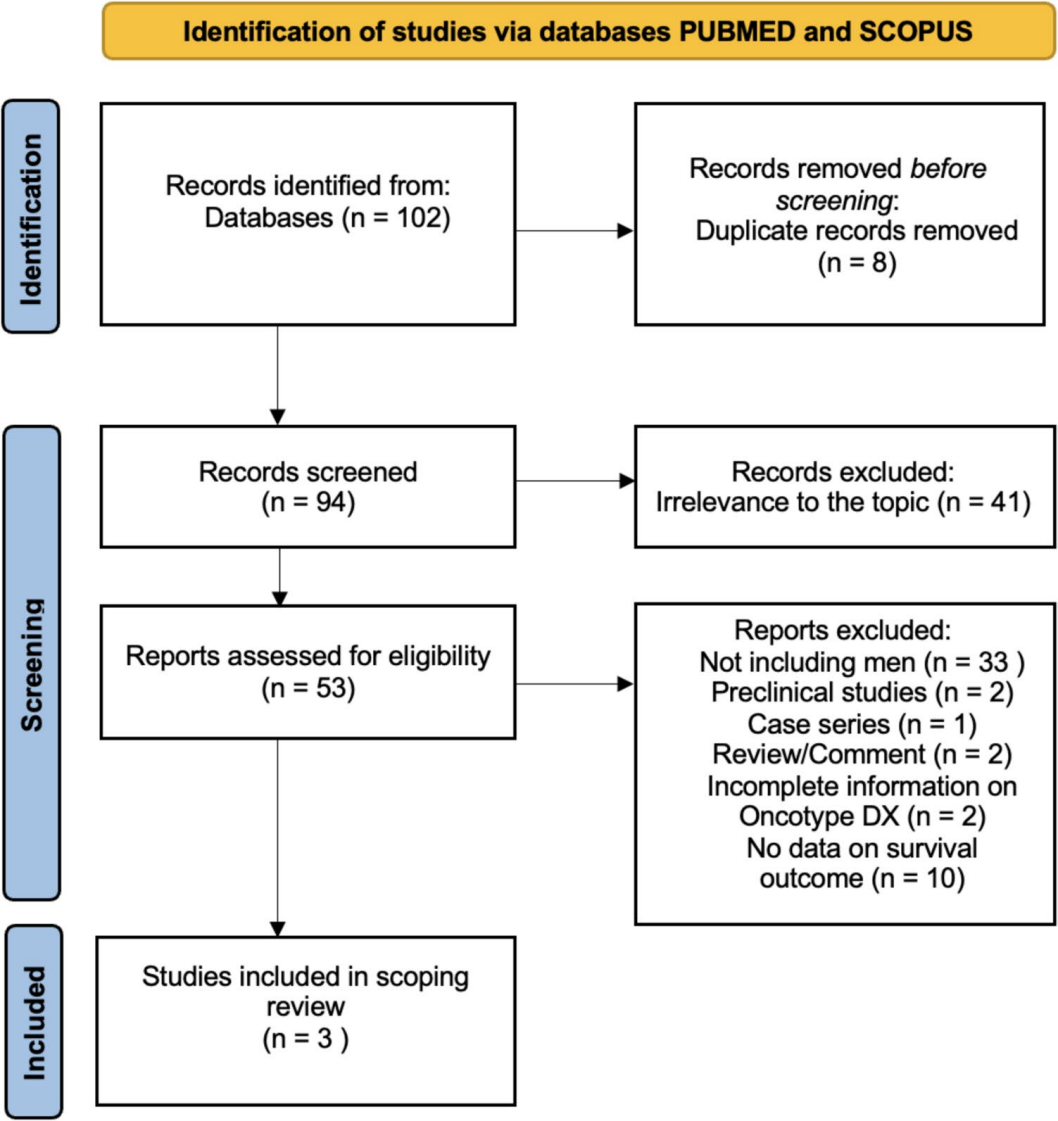
PRISMA flow diagram reporting the study inclusion process

Figure 1 illustrates the article selection process. A total of 102 records were identified in the PubMed and Scopus databases, of which 8 duplicate records were removed before screening. Of the 94 records initially screened, 41 were excluded after abstract evaluation due to irrelevance to the topic. The 53 selected abstracts were thoroughly assessed for eligibility. A total of 50 papers did not meet the inclusion criteria and were excluded from subsequent analyses. A total of three studies on RS data, defined by a 21-gene RT-PCR, performed in male patients reporting outcome measures were retrieved in the final analysis.

### Study characteristics and results

The main characteristics of the three studies included in the analysis are reported in Table 1. All were retrospective observational studies conducted in the USA. One study utilized data from a single institution, while the other two were based on data from large datasets from the SEER database and the National Cancer Database. Outcomes were measured heterogeneously across the studies, including DFS, breast cancer-specific survival (BCSS), and OS.

**Table 1:** Characteristic of included studies

*N*	Study	Country	Study type (years)	Study population	Outcome measures
[28]	Turashvili G et al. 2018	USA	Retrospective (2006–2016)	38 men with breast cancer from a single institution	Disease free survival (DFS)
[29]	Massarweh SA et al. 2018	USA	Retrospective (2004–2017)	3,806 men and 571,115 women with breast cancer with survival data from the SEER database*	5-year breast cancer-specific survival (BCSS) and overall survival (OS)
[30]	Wang F et al. 2020	USA	Retrospective (2010–2014)	848 men and 110,898 women diagnosed with breast cancer from the National Cancer Database	5-year overall survival (OS)

_*_Survival data available for a subset of BC cases of 322 men and 55,842 women

#### Inclusion criteria and clinical-pathologic characteristics of MBC patients

*Turashvili *et al*.* aimed to assess the association between the RS, type of treatment, and outcomes in MBC. Their study included 38 ER-positive, HER2-negative, N0 or a few lymph node-positive MBC patients. Data on clinical and pathological features and treatment were also collected, including age at diagnosis, *BRCA* mutation status, past oncologic history, tumor type and size, lymph vascular invasion (LVI), type of local and systemic treatment, and instances of distant and locoregional recurrence [28].

*Massarweh *et al*.* aimed to evaluate correlations between the RS and BCSS and OS in male patients, comparing the results with those from the female population. Their study included 3,806 men and 571,115 women with ER-positive and/or progesterone receptor (PR)-positive, HER2-negative, N0, microscopic positive (N1mi), or positive with 1 to 3 lymph nodes (N1, 1–3 LN) BC who underwent Genomic Health RS testing. Other clinicopathologic features collected included age at diagnosis and histology. Survival data were retrieved for a subset of 322 male and 55,842 female BC patients from the SEER database [29].

*Wang *et al*.* aimed to assess the association between the RS and mortality in male patients, comparing the results with those from the female population. Their study included 848 men and 110,898 women with ER-positive, HER2-negative, stage I and II breast cancer who underwent surgery. Detailed demographic and clinical-pathologic data were collected, including age at diagnosis, ethnicity, year of diagnosis, tumor size, nodal status, PR status, histology type, histologic grade, LVI, and Charlson/Deyo score. Data on the receipt of treatment, including breast surgery, chemotherapy, radiotherapy, and endocrine therapy, were also collected [30].

#### RS risk categories and cutoffs in MBC

**Fig. 2:**
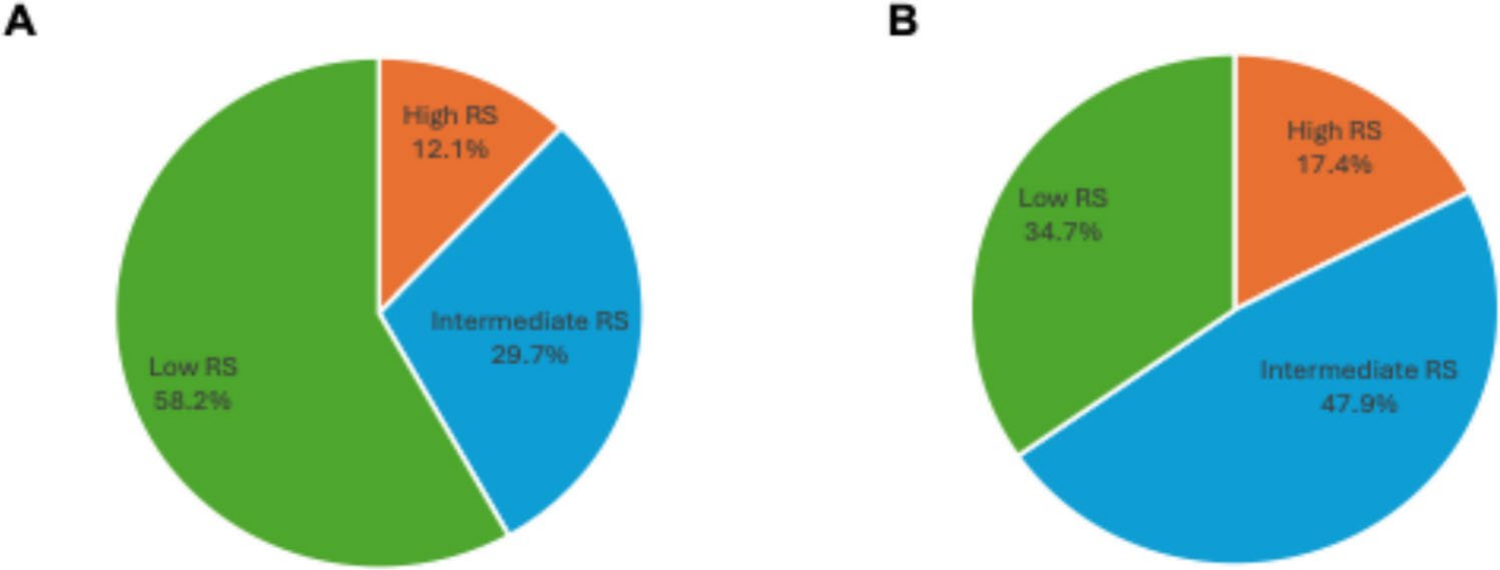
Pie chart illustrating the distribution of Recurrence Scores based on: **A** traditional cutoffs in 4,692 MBC cases from the 3 studies reported in Table 1; and **B** TAILORx cutoffs in 848 MBC cases from the study by *Wang* et al.

All BC cases included in the three studies had available 21-gene RS assay results. All three studies stratified patients into low-, intermediate-, and high-risk groups based on traditional RS cutoffs (< 17, 18–30, and ≥ 31) [18, 19]. The most recent study by *Wang* et al. also stratified patients based on the new TAILORx cutoffs (≤ 10, 11–25, and ≥ 26) [21, 22]. Of the 38 male BC cases reported by *Turashvili *et al., 26 (68.4%) had low RS, 9 (23.7%) had intermediate RS, and 3 (7.9%) had high RS [28]. *Massarweh* et al. reported low RS in 2,207 (58%) MBC cases, intermediate RS in 1,126 (29.6%), and high RS in 473 (12.4%) MBCs. *Wang *et al*.* reported low RS in 496 (58.5%) male BC cases, intermediate RS in 260 (30.7%), and high RS in 92 (10.8%) MBCs. Considering the merged cohort of 4,692 male BC cases from the 3 studies, the RS distribution was 58.2% low RS, 29.7% intermediate RS, and 12.1% high RS (Fig. 2) [29]. Based on the TAILORx cutoffs from *Wang *et al*.*, the distribution was 34.7% low RS, 47.9% intermediate RS, and 17.4% high RS [30] (Fig. 2).

*Turashvili *et al*.* reported no significant association between the RS risk category and any of the clinicopathologic variables analyzed, treatment, or outcomes, likely due to the small sample size [28]. The median follow-up reported was 34 months (range 1–133 months); the patient with the longest follow-up was in the low-risk group (RS = 4). One patient with an intermediate-risk RS developed distant metastasis at 29 months of follow-up, while other patients showed no evidence of disease at follow-up, regardless of their RS group. It was also reported that one patient died at 2 years of follow-up due to other causes. Despite the well-characterized series, the reported analyses were only descriptive; thus, the results from this study will not be considered further in this scoping review.

In the univariate analysis, RS based on traditional cutoffs was associated with both 5-year OS and BCSS, using the log-rank test. The 5-year BCSS was 99.0%, 95.9%, and 81.0% for men in the low-, intermediate-, and high-risk RS groups, respectively (log-rank p = 0.003) [29]. *Massarweh *et al. and *Wang* et al. reported 5-year OS ranging from 92.6% to 95.7% for men in the low-risk RS group, from 86% to 87.5% for men in the intermediate-risk RS group, and from 69.9% to 82.3% for men in the high-risk RS group (log-rank *p* = 0.02 in both studies) [29, 30]. *Wang *et al*.* also analyzed the 5-year OS based on TAILORx cutoffs, reporting an OS of 97.2% for men with RS ≤ 10, 91% for men with RS 11–25, and 83.2% for men with RS ≥ 26 (log-rank *p* = 0.003) [30].

#### Comparison of oncological outcomes between MBC and FBC according to RS risk categories

A comparative analysis of oncological outcomes stratified RS categories between MBC and FBC was conducted based on the data from *Massarweh* et al. and *Wang *et al. (Table 2). Both studies included in this review reported a higher proportion of MBC patients with high-risk RS (≥ 31) compared to FBC. Specifically, in the study by *Massarweh* et al., 12.4% of male patients had RS ≥ 31 versus 7.4% of females; in *Wang *et al., the figures were 10.8% for males versus 7.9% for females.

**Table 2:** Studies included in the scoping review: oncological outcomes according to risk categories

Author	RS	Male	Female	Cht use, male	Cht use female	5y OS male	5y-OS female	5y BCSS male	5y BCSS female	Median follow-up
*Massarweh* et al. [29]	< 18	58%	58.2%	11.1%	8.4%	92.6%	95%	99%	99.5%	Not reported
	18–30	29.6%	34.4%	31.1%	36%	86%	94.2%	95.9%	98.6%	
	> 31	12.4%	7.4%	66.7%	71%	69.9%	89.9%	81%	94.9%	
*Wang *et al. [30]	< 18	58.5%	59.5%	6.2%	6.7%	95.7	97.1%	–	–	39.5
	18–30	30.7%	32.6%	34.2%	39.3%	87.5	96.1%	–	–	
	> 31	10.8%	7.9%	78.3%	83%	82.3%	91.5%	–	–	
	< 11*	34.7%	23.4%	6.8%	4.4%	97.2%	96.6%	–	–	
	11–26 *	47.9%	62.1%	16.5%	18.5%	91%	97%	–	–	
	> 26 *	17.4%	14.5%	70.9%	74.8%	83.2%	92.9%	–	–	

_*_TAILORx cutoffs

Regarding RS distribution, in the Massarweh cohort, 58.0% of MBC patients and 58.2% of FBC patients were categorized as low risk (< 18), while 29.6% and 34.4% were classified as intermediate risk (RS 18–30), respectively. In the Wang study, 58.5% of men and 59.1% of women were low risk, while 30.7% of men and 32.6% of women were in the intermediate-risk group. The proportion of patients receiving chemotherapy increased with higher RS categories in both sexes across both studies (Table 2).

In terms of survival outcomes, OS and BCSS were compared between men and women. OS was evaluated in both studies, while BCSS was reported only by *Massarweh *et al. Notably, men consistently showed inferior 5-year OS rates compared to women across both intermediate and high RS groups. In the Massarweh study, 5-year OS was 86.0% in men vs. 94.2% in women for intermediate RS, and 69.9% vs. 89.9% for high RS. Similarly, *Wang* et al. reported 5-year OS of 87.5% in men vs. 96.1% in women (intermediate RS), and 82.3% vs. 91.5% (high RS).

BCSS data from *Massarweh *et al. also demonstrated a trend toward worse cancer-specific outcomes in men: in the intermediate-risk group, 5-year BCSS was 95.9% in males versus 98.6% in females, and in the high-risk group, 81.0% in males versus 94.9% in females.

#### Lymph node status

In Massarweh et al*.,* 85.7% of the MBC series was N0 [29]. The distribution of RS risk groups differed based on lymph node status, with RS ≥ 31 in 11.6% of N0 MBC and in 21.7% of N1mi, 1-3LN cases. Moreover, analyses stratified by lymph node status showed that both 5-year BCSS and OS estimates were significantly different between RS groups based on traditional cutoffs in men with N1mi and N1-3 breast cancer (log-rank BCSS *p* = 0.02; OS *p* = 0.002) but not in men with N0 breast cancer (log-rank BCSS *p* = 0.08; OS *p* = 0.22).

In Wang et al*.,* 81% of the MBC series was N0. Lymph node status was considered in a multivariate Cox regression model that included additional adjustments for demographic characteristics, clinical characteristics, and treatments, with the exception of chemotherapy [30]. In this model, based on TAILORx cutoffs but not on traditional cutoffs, a higher mortality risk was reported for male patients with intermediate-risk RS (HR 5.37; 95% CI, 1.79–16.11) and high-risk RS (HR 4.28; 95% CI, 1.22–14.97) compared with those with low-risk RS. In a sensitivity analysis restricted to patients with negative lymph nodes and receipt of endocrine therapy, results showed a similar trend; however, due to the few deaths in the high-risk RS group, the association between high-risk RS and mortality was not statistically significant.

#### Age

Differences in RS distribution among age groups were observed [29]. In particular, RS ≥ 31 was more frequent in men younger than 40 years of age. Conversely, low RS results (RS < 11) were more common (> 30% of cases) in men over 50 years of age. Only one of the three studies conducted a Cox regression multivariate analysis with adjustment for age [30]. RS was associated with mortality based on both traditional cutoffs (intermediate-risk HR 2.75, 95% CI 1.38–5.49; high-risk HR 3.06, 95% CI 1.28–7.32, compared with low-risk RS) and TAILORx cutoffs (intermediate-risk HR 4.60, 95% CI 1.59–13.32; high-risk HR 7.15, 95% CI 2.35–21.72, compared with low-risk RS). Due to the small number of male patients aged < 50, it was not possible to test the reported interactions between TAILORx cutoffs and age (< 50 years vs. > 50 years). In age-specific analyses conducted in male patients older than 50 years, HRs for total mortality associated with the RS categories were similar to those reported for the entire case series.

#### Adjuvant chemotherapy treatment

Chemotherapy utilization increased with higher RS; in* Wang* et al., 70.9% of men with RS ≥ 26 received chemotherapy, while in *Massarweh *et al., 66.7% of men with RS > 31 received chemotherapy. Adjustment for chemotherapy resulted in little attenuation of the HR for the intermediate-risk group (HR 5.33; 95% CI, 1.77–16.08), but there was some attenuation for the high-risk group (HR 3.56; 95% CI, 0.93–13.58) [30]. No significant association between chemotherapy and mortality was observed among male patients with intermediate-risk RS, using either traditional cutoffs (HR 1.37; 95% CI, 0.32–5.82) or TAILORx cutoffs (HR 3.50; 95% CI, 0.98–12.53), although these analyses were based on a much smaller sample size. Similar results were obtained when restricting analyses to patients with negative lymph nodes who received endocrine therapy.

### Risk of bias in studies

**Fig. 3:**
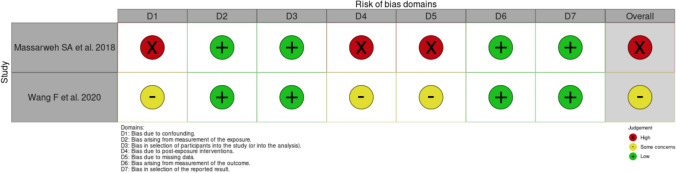
Traffic light plot representing the results from the quality assessment using the ROBINS-E tool

The three studies included in the analysis were assessed using the ROBINS-E tool. Based on the preliminary questions, we decided not to proceed with a risk-of-bias assessment for the study by *Turashvili *et al., as it reported neither unadjusted nor adjusted analysis results, which categorizes it as being at very high risk of bias. The quality assessment of the two papers by *Massarweh *et al. and *Wang *et al*.* is presented in Fig. 3.

### Synthesis of results

The inclusion criteria and the RS distributions based on traditional cutoffs were highly consistent across studies. In univariate analyses, both the traditional and TAILORx RS thresholds were significantly associated with clinical outcomes, with notably higher mortality observed in high-risk MBC patients. However, among patients without nodal involvement, the association between RS groups, based on traditional cutoffs, and outcomes lost statistical significance.

After adjustment for demographic and clinical characteristics, including lymph node status and treatments other than chemotherapy, the intermediate- and high-risk categories defined by TAILORx, but not by traditional cutoffs, remained significantly associated with increased mortality in men.

Although some differences in RS distribution were observed across age groups, age-specific analyses could not be performed due to the small number of male patients under 50 years of age.

While the RS demonstrated evident prognostic value in MBC, even in the context of adjuvant chemotherapy, its capacity to predict chemotherapy benefit appears limited. Importantly, current data do not allow for a clear evaluation of the incremental benefit of adjuvant chemotherapy within RS categories in men.

Comparative analyses of oncological outcomes between MBC and FBC patients, stratified by RS risk groups, revealed that despite comparable RS distributions and treatment strategies, MBC patients may experience poorer outcomes, particularly within the higher risk categories. These findings underscore the need for sex-specific validation of genomic assays such as  ODx.

## Discussion

This scoping review identified only three eligible studies evaluating oncologic outcomes in MBC based on the 21-gene ODx RS [28–30]. *Turashvili* et al. presented a small, descriptive series of 38 patients with limited follow-up and event rates, precluding statistical analysis [28]. Consequently, our focus centered on the two larger retrospective cohort studies by *Massarweh *et al. and *Wang* et al. [29, 30].

Both studies confirmed the prognostic value of RS in MBC, demonstrating significant associations between RS categories and OS, regardless of adjuvant therapy. However, the predictive value of RS for chemotherapy benefit in men remains inconclusive.

While RS distributions were broadly similar between MBC and FBC [37], a higher proportion of men exhibited RS ≥ 31, aligning with more advanced disease at diagnosis (larger tumors, greater nodal involvement) and potentially reflecting diagnostic delays or selection bias toward molecular testing in more aggressive cases [25–27, 29, 30, 38–41].

Both studies reported significant associations between RS and survival, with 5-year OS varying across risk categories. In MBC, survival differences were more pronounced, particularly in intermediate- and high-risk groups. Only *Massarweh *et al. evaluated BCSS, showing a trend toward worse outcomes in men, especially in the higher RS categories [29]. *Wang* et al.’s use of 5-year OS, without distinguishing cancer-specific mortality, limits interpretability [30]. Moreover, survival deterioration was steeper in men transitioning from low to intermediate risk, while in women, survival gradients were more gradual. These findings suggest that men generally have poorer outcomes, especially in intermediate/high-risk groups, though confounding by age and comorbidities is likely. In both studies, men were older and had more comorbidities than women [29, 30].

*Wang* et al. observed that mortality risk in men increased with RS values > 21 before plateauing, while in women, risk increased from RS > 23 [30]. These findings imply that current RS cutoffs, established in female populations via TAILORx, may not be optimal for men. In FBC, high RS (≥ 26) was associated with a twofold increase in mortality (HR 2.05), whereas no significant difference was observed between intermediate- and low-risk groups. In contrast, in MBC, mortality rose markedly from low to intermediate risk.

Notably, node-negative MBC patients, representing the majority in both cohorts, did not exhibit significant prognostic stratification by RS. However, node-positive MBC patients did, with higher RS > 31 proportions in this subgroup. In *Massarweh* et al*.,* 5-year BCSS and OS diverged significantly by RS category in node-positive, but not node-negative, MBC patients. In contrast, FBC outcomes retained prognostic value across RS categories irrespective of nodal status. Moreover, the distribution of high RS (> 31) was higher in node-positive MBC (21.7%) versus node-negative MBC (11.6%), unlike FBC where RS > 31 rates were similar across nodal strata [29]. *Wang* et al.’s multivariate analysis, adjusted for lymph node status and other clinical factors, confirmed elevated mortality risk in intermediate/high RS categories [30]. However, the predictive role of RS for chemotherapy benefit in MBC, especially node-positive disease, remains unproven, unlike in FBC where RXponder validated chemotherapy benefit in certain N1-3 cases [24].

Age-specific analyses were limited due to the small number of younger male patients. *Wang* et al. found RS retained prognostic value regardless of age, contrasting with FBC, where chemotherapy benefit is influenced by menopausal status [24]. *Massarweh* et al. noted more high RS results in men < 40 years, yet similar rates of very low RS in both sexes at this age [29]. The absence of *BRCA1/2* status limits interpretation. *BRCA2*-related MBCs are associated with more aggressive phenotypes and higher RS, suggesting genetic influences warrant further exploration [7, 8, 42].

Although MBCs are often ER-positive and viewed as phenotypically homogeneous, transcriptional profiling suggests the presence of distinct molecular subtypes [10, 12]. *Massarweh* et al. reported higher expression of ER- and proliferation-related genes in men versus women, with age-related variation also observed [29].

*Wang* et al. evaluated chemotherapy-adjusted mortality risk across RS categories in MBC. Adjustments resulted in minimal attenuation of hazard ratios, suggesting limited chemotherapy benefit [30]. However, data limitations, including retrospective design and database inaccuracies regarding treatment adherence and regimens, undermine this conclusion. In women with intermediate RS, a modest chemotherapy benefit was noted only for those < 50 years, consistent with TAILORx [22, 23]. In men, no chemotherapy benefit was detected in intermediate RS cases.

This may reflect distinct tumor biology, absence of hormone-suppressive effects seen in premenopausal women, or inherent resistance of luminal tumors to chemotherapy. Luminal A tumors, which predominate in MBC, are less responsive to chemotherapy than luminal B, HER2-positive, or triple-negative tumors [2, 19, 20, 43–45]. Dose-dense chemotherapy provides long-term benefit in node-positive luminal BC, especially where endocrine transcriptional activity is low [42].

Additional insights are provided by *Bayani* et al., who retrospectively analyzed 381 MBC cases using a custom Nanostring panel mimicking commercial genomic tests [46]. While all genomic signatures showed prognostic value in univariate analyses, significance was lost after adjustment, likely due to limited event rates. Their results, consistent with those of *Massarweh *et al., reinforce RS’s prognostic utility but were excluded from our review due to the computational derivation of RS rather than use of the commercial assay.

The primary limitations of this scoping review include the small number of studies, their retrospective nature, and incomplete data on *BRCA* status and treatment specifics. Only *Wang* et al. applied both traditional and TAILORx RS cutoffs, complicating comparison with *Massarweh *et al., who used only the traditional cutoffs. Furthermore, OS may be confounded by non-cancer deaths, especially in older MBC patients with higher comorbidity burdens.

Nonetheless, this review provides important insights. It shows the prognostic relevance of RS in MBC and underscores the need for RS categorization tailored to men to avoid under- or overtreatment. It also highlights the importance of enrolling men in treatment studies and conducting molecular profiling to better understand disease biology and guide personalized therapy.

## Conclusion

Differences in survival patterns, limited predictive value of RS for chemotherapy, and the influence of age and nodal status indicate that sex-specific RS thresholds may be necessary for optimal treatment stratification in MBC. Given the rarity of MBC and the limitations of current data, prospective, male-inclusive trials and comprehensive genomic studies are urgently needed. Until then, clinicians should interpret RS in men cautiously, within a multidisciplinary framework that considers tumor biology, comorbidities, and patient preferences.
